# Supplementary material for: Causal Relationship between Aging and Anorexia Nervosa: A White-Matter-Microstructure-Mediated Mendelian Randomization Analysis
Source: Biomedicines. 2024 Aug 16;12(8):1874. doi: 10.3390/biomedicines12081874 (PMC11351342; doi:10.3390/biomedicines12081874)
Supplement: Supplementary file 1 [file biomedicines-12-01874-s001.zip › Supplementary Figure/Figure S4/Singe SNP plot of MR analysis for TL to ubm-b-1994.pdf]

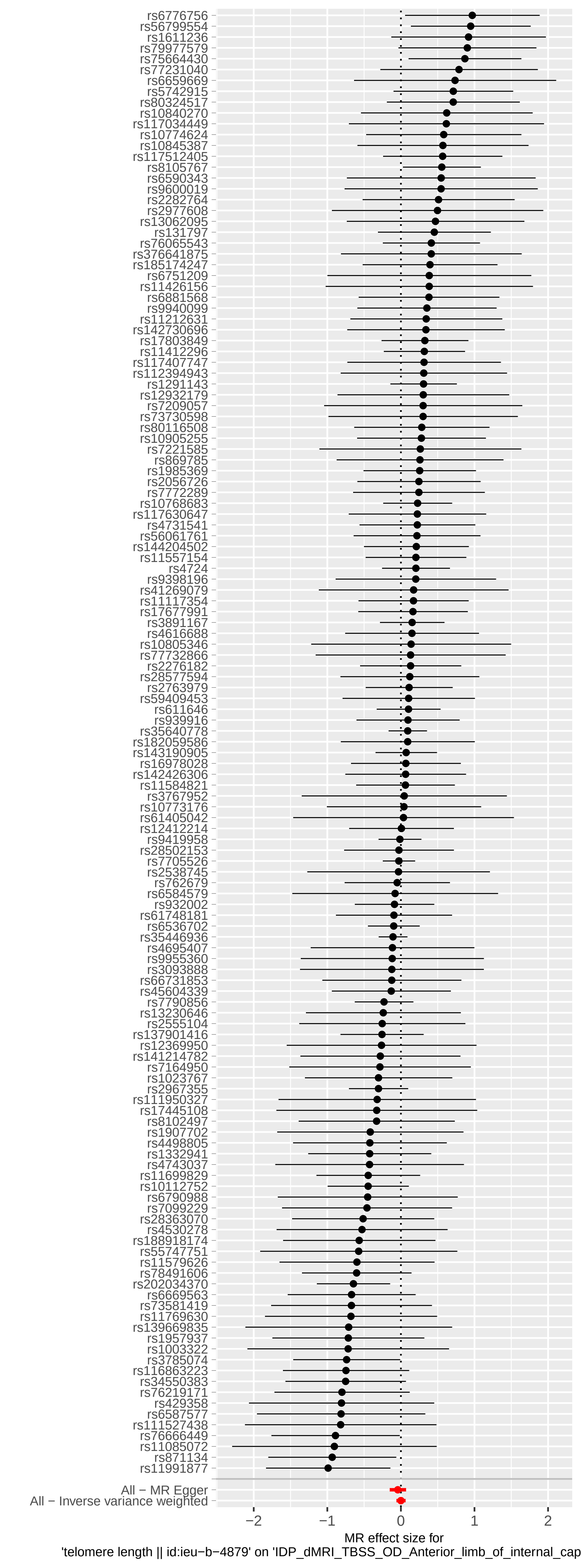

rs6776756
rs56799554
rs1611236
rs79977579
rs75664430
rs77231040
rs6659669
rs5742915
rs80324517
rs10840270
rs117034449
rs10774624
rs10845387
rs117512405
rs8105767
rs6590343
rs9600019
rs2282764
rs2977608
rs13062095
rs131797
rs76065543
rs376641875
rs185174247
rs6751209
rs11426156
rs6881568
rs9940099
rs11212631
rs142730696
rs17803849
rs11412296
rs117407747
rs112394943
rs1291143
rs12932179
rs7209057
rs73730598
rs80116508
rs10905255
rs7221585
rs869785
rs1985369
rs2056726
rs7772289
rs10768683
rs117630647
rs4731541
rs56061761
rs144204502
rs11557154
rs4724
rs9398196
rs41269079
rs11117354
rs17677991
rs3891167
rs4616688
rs10805346
rs77732866
rs2276182
rs28577594
rs2763979
rs59409453
rs611646
rs939916
rs35640778
rs182059586
rs143190905
rs16978028
rs142426306
rs11584821
rs3767952
rs10773176
rs61405042
rs12412214
rs9419958
rs28502153
rs7705526
rs2538745
rs762679
rs6584579
rs932002
rs61748181
rs6536702
rs35446936
rs4695407
rs9955360
rs3093888
rs66731853
rs45604339
rs7790856
rs13230646
rs2555104
rs137901416
rs12369950
rs141214782
rs7164950
rs1023767
rs2967355
rs111950327
rs17445108
rs8102497
rs1907702
rs4498805
rs1332941
rs4743037
rs11699829
rs10112752
rs6790988
rs7099229
rs28363070
rs4530278
rs188918174
rs55747751
rs11579626
rs78491606
rs202034370
rs6669563
rs73581419
rs11769630
rs139669835
rs1957937
rs1003322
rs3785074
rs116863223
rs34550383
rs76219171
rs429358
rs6587577
rs111527438
rs76666449
rs11085072
rs871134
rs11991877
All − MR Egger
All − Inverse variance weighted
−2
−1
0
1
2
MR effect size for
'telomere length || id:ieu−b−4879' on 'IDP_dMRI_TBSS_OD_Anterior_limb_of_internal_cap
